# Color, anthocyanin, and antioxidant characteristics of young wines produced from spine grapes (*Vitis davidii* Foex) in China

**DOI:** 10.1080/16546628.2017.1339552

**Published:** 2017-07-14

**Authors:** Fuliang Han, Yanlun Ju, Xianrui Ruan, Xianfang Zhao, Xiaofeng Yue, Xifu Zhuang, Minyang Qin, Yulin Fang

**Affiliations:** ^a^ College of Enology, Northwest A&F University, Yangling, China; ^b^ Quality Supervision Department, Gentleman Valley Wild Fruits World Co. Ltd., Chongyi, Jiangxi, China

**Keywords:** Wild grape, wine, color, anthocyanins, antioxidant activity

## Abstract

**Background**: Spine grape has gained attention in the field of wine science due to its good growth characteristics. Spine grape wine has been made by local residents for a long time. However, the scientific evaluation of spine wine has not been systemically documented compared to *Vitis vinifera* grape wines

**Methods**: We compared 11 spine wines from south China (W1–W11) with 7 high-quality international wines (W12–W18). The total phenolic content, the total anothcyanin content and the antioxidant activity of these wines were analyzed and compared. Meanwhile, anthocyanin profiles of these wines were also documented.

**Results**: Compared with other wines most of the spine wines had a strong red intensity with a blue hue. Malvidin-3,5-*O*-diglucoside and malvidin-3-*O*-(6-*O*-coumaroyl)-glucoside-5-glucoside appeared to be the major anthocyanins in these wines. The scavenging capacity analyses of these wines using ABTS, DPPH, and CUPRAC assays indicated that spine wines possessed high antioxidant properties, especially spine wine W3, W4, W6 and W8. Their high antioxidant properties were mainly related to the high levels of the total phenolic content and anthocyanins.

**Conclusion**: These results suggested that spine wine might be considered a good wine source for the Chinese wine industry and provided useful information on the knowledge of spine grape.

##  Introduction 

Phenolic compounds can be classified into anthocyanin and non-anthocyanin phenolics [1]. Phenolic compounds possess a number of bioactive functions, such as antioxidant, cardiovascular protective, anticancer, and anti-inflammatory properties [2–5]. Besides, they are the major components in wine that play an important role in contributing the sensory attributes and mouthfeel to wine [6,7]. It has been accepted that red wine can be firstly attracted to consumers via its color, and that customers have a preference for wines with an attractive color and good nutritional values [8,9]. Therefore, it is critical to produce red wine with nice color and high nutritional properties.

**Figure 1.**
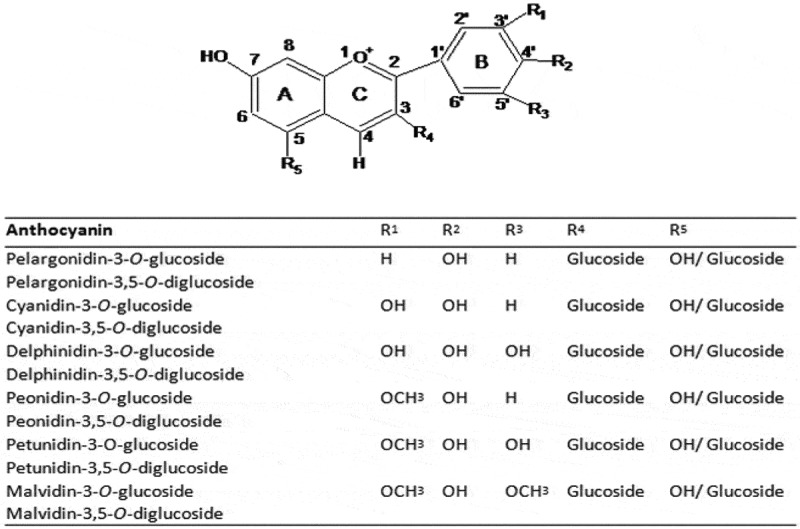
Structure of anthocyanin monoglucoside and diglucosides in red wine.

Anthocyanins are important pigments in red wine. Based on different substitutions in their B-ring, aglycones mainly include pelargonidin, cyanidin, delphinidin, peonidin, petunidin, and malvidin (Figure 1). It has been known that *Vitis vinifera* grapes have anthocyanidin-3-*O*-monoglucoside as the major anthocyanins, whereas diglucoside conjugated anthocyanins are mainly present in other grapes, such as *V. amurensis, V. riparia, V. rupestris*, and their hybrids [10–13]. Besides, anthocyanins can further be metabolized to yield acylated anthocyanins, pyranoanthocyanins, and polymeric anthocyanins. It has been confirmed that malvidin mono- or diglucosides and their derivatives appear to be the predominant anthocyanins in wines [10–14].

Phenolic compounds have been reported to have capacity of scavenging free radicals, donating hydrogen, chelating metal ions, breaking radical chain reaction, and quenching singlet oxygen [15]. It has been also suggested that phenolic compounds could enhance the endogenous antioxidant capacity of cells/tissues and interact with cellular receptors and/or enzymes, which could improve human health [16]. There are many *in vitro* methods that have been used to determine the antioxidant properties of phenolic compounds [15,17,18]. For example, 2,2ʹ-azinobis-3-ethylbenzothiazoline-6-sulfonic acid (ABTS) and 2,2-diphenyl-1-picrylhydrazyl (DPPH) are two free radicals that can be scavenged with the presence of antioxidants, and therefore ABTS and DPPH assays can estimate antioxidant capacity of phenolic compounds [17,18]. Antioxidants in cupric reducing antioxidant capacity (CUPRAC) assay can convert neocuproine/copper (II) complex to neocuproine/copper (I) complex, which results in a change on the complex absorption characteristics [19]. It has been confirmed that antioxidant features of phenolic compounds in wine are mainly depended on its variety, origin and vintage [15,20,21].

The major regions for spine grape growth in China are located in the Yangtze River basin and the Yunnan–Guizhou plateau [22,23]. This grape species possesses the properties of strong vigor, climate adaptation, disease resistance, high humidity resistance, and low light resistance [23–25]. Spine wine has been generally produced by the local residents for a long time. However, the scientific evaluation of spine wine has not been systemically documented in terms of phenolic content, anthocyanin composition and antioxidant activity compared to *V. vinifera* grape wines [26–28]. In this study, we selected 11 spine wine samples compare with other seven conventional wines. This study could provide useful information on the knowledge of spine grape and wine, which could enhance the market growth of spine wine in China.

## Materials and methods

### Chemicals

Malvidin-3-*O*-glucoside and gallic acid were purchased from Sigma-Aldrich (Shanghai, China) with a purity of 95.0% and 97.5%, respectively. ABTS, DPPH, neocuproine, and 6-hydroxy-2,5,7,8-tetramethylchroman-2-carboxylic acid (Trolox) were obtained from Sigma-Aldrich (Shanghai, China). Formic acid and acetonitrile were of HPLC grade and purchased from Anpel Company (Shanghai, China) and Tedia (Fairfield, USA), respectively. All other chemicals used in the study were of analytical grade.

### Wine samples

Eleven spine wine samples (W1–W11) were collected from Tongmu winery (Hu’nan Province, China; 27°29′N, 110°31′E, mean altitude: 140 m, average precipitation: 1378 mm, subtropical monsoon climate, soil type: limestone). Seven conventional wines, including three rosé wines (W12: Yantai, China; W13: Bordeaux, France; W14: Provence, France), two white wines (W15: Bordeaux, France; W16:Napa Valley, USA), and two red wines (W17: Bordeaux, France; W18: Napa Valley, USA), were bought from a wine market in Beijing, China. The wine sample W1 (rosé wine) was directly fermented from grape juice without maceration. The rest of the wine samples were mixed with 50 mg/L SO_2_ and then macerated for 6 days after the addition of 0.02 g/L pectinase. After the maceration, 0.2 g/L active dry yeast was added to initiate alcoholic fermentation. The grapes used for the wine sample W8 were harvested from a vineyard where Xiangzhenzhu1^#^, Xiangzhenzhu2^#^, and Xiangzhenzhu3^#^ grape strains were grown in a combined cultivation way. The percentage of each grape strain was not specified. The appropriate sugar content was added to the grapes during fermentation in order to achieve the required alcoholic content. The fermentation temperature was controlled at 24–30°C and the fermentation was accomplished within 7 days. The malolactic fermentation took place without inoculation in all wines except for the wine sample W4. All the wine samples were stored at −20°C prior to further analyses.

### Total phenolic content measurement

Total phenolic content measurement followed a published method with minor modifications [21]. Briefly, the wine sample (0.1 mL) was mixed with 0.5 mL of Folin–Ciocalteu reagent and 2 mL of 20 g/L sodium carbonate solution. The resultant mixture was further diluted with distilled water to 10 mL. This resulting reaction solution was incubated at room temperature for 60 min and then its absorbance was measured at 765 nm. The total phenolic content was expressed as gallic acid equivalent (GAE mg/L).

### Wine color analysis

Wine color analysis was carried out on a UV-2450 spectrophotometer (Shimadzu, Japan) according to the published methods [29,30]. Briefly, the transmittance of the wine sample was measured at 440, 530, and 600 nm using a 0.2 cm path-length quartz cuvette. Before the measurement, the wine sample was filtered through 0.45 µm membranes. Distilled water was used as the blank. The values of L*, a*, and b* were calculated using the llluminant D65 and 10° observer angle. The chroma was expressed as C = (a*^2^ + b*^2^)^1/2^, whereas the tone was expressed as H = arctan(b*/a*). The color difference was expressed as ΔE = [(ΔL)^2^ + (Δa*)^2^ + (Δb*)^2^]^1/2^. The spine wine sample W1 (rosé wine) was used as the reference.

### Anthocyanin quantitation

LC-20AT HPLC system (Shimadzu, Japan) was used for the analysis of anthocyanins according to our published paper [6,31]. An Agilent SB-C18 column (250 × 4.6 mm^2^, 5 µm, Santa Clara, CA, USA) was used for the separation of anthocyanins with a flow rate of 1 mL/min. The injection volume was 20 µL and the column temperature was maintained at 35°C. The mobile phase consisted of (A) 2% formic acid in water and (B) acetonitrile. The gradient program was set as follows: 0–30 min, 0–20% B; 30–45 min, 20–35% B; 45–46 min, 35–100% B; 46 51 min, isocratic 100% B; 51–52 min, 100% to 0% B; and 52–57 min, isocratic 0% B. Malvidin-3-*O*-glucoside was used as the external standard for quantitation of individual anthocyanins. An Accela 600 HPLC system coupled with a Thermo Fisher LTQ XL ion trap mass spectrometer (Thermo Fisher Scientific Inc, San Jose, CA, USA) was used for the identification of anthocyanins in the wine samples based on the published methods [14,23].

### Antioxidant capacity measurements

#### ABTS radical scavenging activity

ABTS radical scavenging activity of the wine sample was performed according to the published methods with minor modifications [15,32]. Briefly, 7 mM ABTS solution was mixed with 140 mM potassium persulfate aqueous solution to generate ABTS radical cation. The resulting mixture was kept in the dark at room temperature for 12 h. Before the measurement, the resultant ABTS solution was diluted with ethanol to an absorbance of 0.70 ± 0.02 at 732 nm. The wine sample was diluted at 1:30 using 15% (v/v) ethanol and then 0.1 mL diluted wine sample was mixed with 3.9 mL of the diluted ABTS solution in the dark for 8 min at room temperature. Afterwards, the absorbance of the resultant solution was measured at 732 nm. The 15% (v/v) ethanol (0.1 mL) mixed with 3.9 mL of the diluted ABTS solution was used as the control. Trolox was used as the external standard (50–800 µM) and the result was expressed as Trolox equivalent antioxidant capacity (TEAC).

### DPPH radical scavenging activity

0.1 mL of the diluted wine sample was mixed with 3.9 mL of 2.5 mg/L DPPH solution in the dark for 20 min at room temperature. Afterwards, the absorbance of the sample was measured at 517 nm [15,33]. The same volume of 15% ethanol solution mixed with 3.9 mL of 2.5 mg/L DPPH solution was used as the control. Trolox was used as the external standard with the concentration of 50–1000 µM. The result was expressed as TEAC.

### CUPRAC assay

The CUPRAC measurement followed two published methods with some modifications [34]. Briefly, 1 mL of 5 mM copper sulfate, 3.75 mM neocuproine, 1 M ammonium acetate buffer (pH 7.0), and 2.9vmL of deionized water were mixed. The resultant solution was mixed with 0.1 mL of the diluted wine sample. The reaction was conducted for 30 min in the dark at room temperature, and then the absorbance of the sample was recorded at 450vnm. 0.1vmL of 15% (v/v) ethanol mixed with the same volume of the reacting solution was considered the control.

### Statistical analysis

Each wine sample was analyzed in triplicate tests. SPSS 22.0 statistical software was used to perform the statistical analysis, including the analysis of variance (ANOVA), Tukey’s multiple range tests with a significance level at 0.05, a two-tailed Pearson’s correlation test and partial least square regression (PLSR). The Pearson’s correlation test was used to determine the correlation between antioxidant capacity and phenolic compounds. PLSR was used to investigate the contribution of individual anthocyanins to the wine color attribute [27].

## Results and discussion

### Color attribute of spine wines

It was observed that the ΔE values of all the tested wines in the present study were greater than 1 (Table 1), indicating that these wines had the differences in their color [35]. CIELAB color space is generally used to analyze wine color features, such as color intensity, chroma, hue, etc. In this study, spine rosé wine W1 showed the lowest H value among all wine samples, suggesting that it possessed the strongest blue hue. The L* value of rosé wine sample W1 was higher than that of rosé wine samples W12, W13, and W14. Rosé wines W12, W13, and W14 showed less red color (lower a* value), more yellow color (higher b* value and H value), and weaker chroma (lower C value). In red wines, W3, W4, and W8 had dark color due to their lower L* value, while W17 and W18 presented the similar L* value compared with W6, W7, W10, and W11. The red color of W17 and W18 was weaker than other spine grape wines except W9, but they presented the strongest yellow color (higher b* value). Their C value and H value also indicated their weaker chroma and stronger yellow tone.

**Table 1.** Color attributes of wines.

Sample	L*	a*	b*	C	H	ΔE
W1	87.22 ± 0.05	20.08 ± 0.01	−6.91 ± 0.04	21.24 ± 0.02	−19.00 ± 19.00	Control
W2	43.60 ± 0.11	61.24 ± 0.06	−4.50 ± 0.07	61.40 ± 0.06	−4.20 ± 0.06	60.02
W3	31.65 ± 0.39	65.04 ± 0.12	−11.24 ± 0.21	66.0 ± 0.16	−9.81 ± 0.16	71.61
W4	33.81 ± 0.53	64.54 ± 0.17	−14.06 ± 0.35	66.06 ± 0.24	−12.29 ± 0.27	69.86
W5	71.63 ± 0.10	43.88 ± 0.19	−5.01 ± 0.10	44.17 ± 0.20	−6.52 ± 0.10	28.52
W6	48.98 ± 0.32	62.92 ± 0.12	−17.07 ± 0.19	65.19 ± 0.17	−15.18 ± 0.13	58.31
W7	58.61 ± 0.10	54.21 ± 0.15	−4.62 ± 0.11	54.41 ± 0.16	−4.87 ± 0.10	44.60
W8	32.83 ± 0.68	64.68 ± 0.22	−12.00 ± 0.41	65.79 ± 0.29	−10.51 ± 0.32	70.52
W9	70.29 ± 0.03	37.87 ± 0.09	2.94 ± 0.03	37.98 ± 0.08	4.44 ± 0.06	26.46
W10	60.60 ± 0.31	57.31 ± 0.33	−7.64 ± 0.20	57.82 ± 0.35	−7.59 ± 0.15	45.78
W11	65.24 ± 0.08	47.97 ± 0.44	−12.23 ± 0.23	49.50 ± 0.48	−14.30 ± 0.13	35.91
W12	90.04 ± 0.06	3.11 ± 0.12	8.42 ± 0.34	8.97 ± 0.13	1.22 ± 0.01	17.27
W13	89.55 ± 0.12	2.58 ± 0.01	10.64 ± 0.32	10.9 ± 0.21	1.33 ± 0.12	18.04
W14	93.71 ± 0.03	−1.09 ± 0.19	5.92 ± 0.22	6.02 ± 0.34	−1.39 ± 0.02	22.16
W17	60.37 ± 0.43	30.30 ± 0.21	25.45 ± 0.01	39.57 ± 0.34	0.70 ± 0.10	34.19
W18	50.18 ± 0.24	36.19 ± 0.09	21.94 ± 0.05	42.32 ± 0.25	0.55 ± 0.02	89.77

The wine sample W1 was used as the control for the ΔE calculation. White wines W15 and W16 were not studied.

According to the value of color parameters, all spine red wines presented different color properties. It should be noted that although the wine samples W5 and W6 were fermented using the same spine grape strain (Xiangzhenzhu2^#^), they significantly differed in their color features. The wine samples W7 and W10 were produced from Xiangzhenzhu and Miputao spine grape strain, respectively. However, they showed the similar lightness and color value due to the similar L* and ΔE values. It was also noted that higher a* and C values were observed in the wine samples W2, W3, W4, W6, W8, and W10 compared to the other spine wine samples, which indicated that these wines had a deeper red color and a brighter chroma. The wine samples W3, W4, W6, W8, and W11 exhibited stronger blue color since they showed lower negative b* and H values, whereas the weaker blue color was observed in the samples W2, W5, W7, and W10.

It has been confirmed that the composition and distribution of phenolic compounds, especially anthocyanins, is the most important parameter to affect color characteristics of wine [6]. Grape variety and origin, grape cultivation technology, winemaking process/technology, and wine aging play important roles in determining the color attributes of final wine [13,26,36,37]. For example, the spine grape wines displayed much deeper color, stronger red intensity, purer chroma, and strong blue hue compared to young red wine made of *V. vinifera* grape varieties [36,37]. It has been generally accepted that deep and bright red color in wine normally has more potential to attract customers before taste [8,9]. Therefore, spine wines might have more potential to appeal wine consumers due to their favorable color characteristics.

### Anthocyanins in spine wines

**Figure 2.**
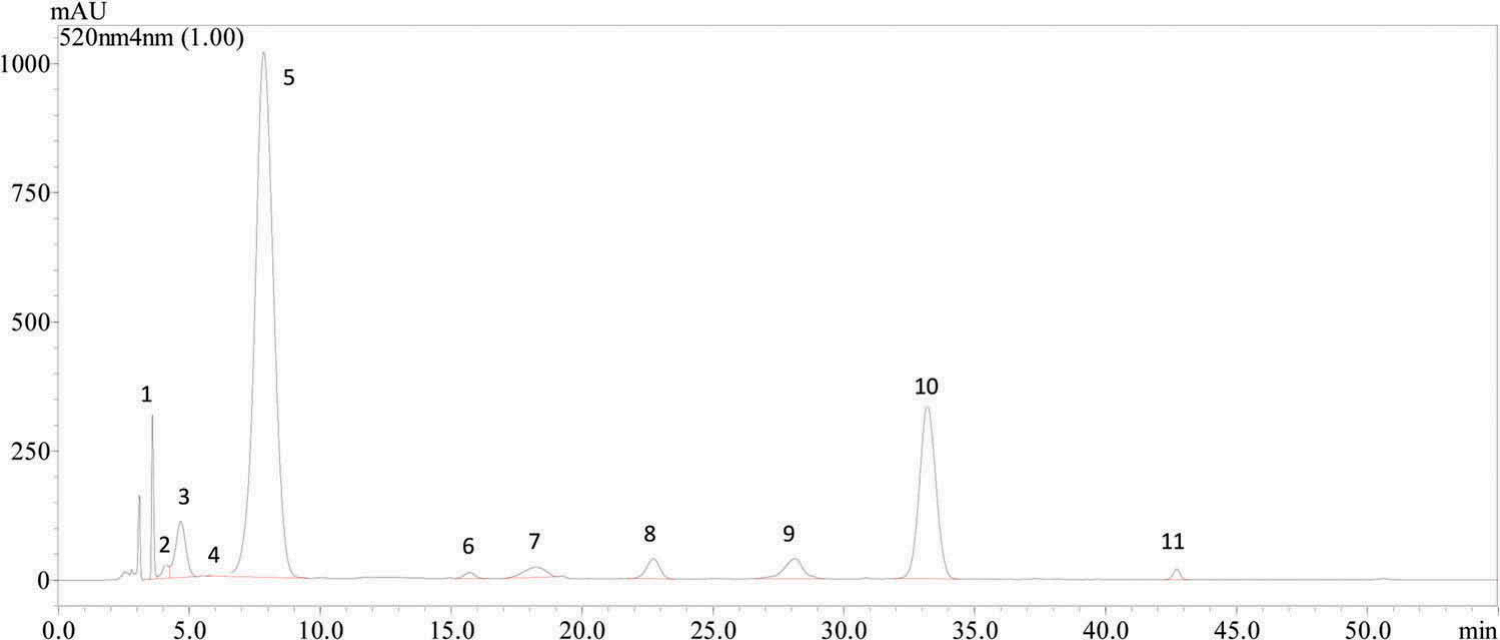
HPLC chromatography of anthocyanins in spine wine made of spine grape strain Xiangzhenzhu1^#^ (W3). Peaks 1, 2, 3, 4, 5, 6, 7, 8, 9, 10, and 11 represent delphinidin-3,5-*O*-diglucoside, cyanidin-3,5-*O*-diglucoside, petunidin-3,5-*O*-diglucoside, peonidin-3,5-*O*-diglucoside, malvidin-3,5-*O*-diglucoside, peonidin-3-*O*-(6-*O*-acetyl)-glucoside-5-*O*-glucoside, malvidin-3-*O*-(6-*O*-acetyl)-glucoside-5-*O*-glucoside, delphinidin-3-*O*-(6-*O*-coumaryl)-glucoside-5-*O*-glucoside, petunidin-3-*O*-(6-*O*-coumaryl)-glucoside-5-*O*-glucoside, malvidin-3-*O*-(6-*O*-coumaryl)-glucoside-5-*O*-glucoside, and malvidin-3-*O*-(6-*O*-coumaryl)-glucoside, respectively.

A total of 11 anthocyanins were detected in spine wines (Figure 2 and Table 2), including five anthocyanidin diglucosides, two acetylated anthocyanidin diglucosides, 3 coumaroylated anthocyanidin diglucosides, and one coumaroylated anthocyanidin monoglucoside. In *V. vinifera* grape wine, its anthocyanin profile is mainly comprised of anthocyanidin monoglucosides with malvidin-3-*O*-glucoside and its derivatives as the predominant anthocyanins [13,27]. In this study, nine anthocyanidin monoglucosides were detected in *V. vinifera* grape red wines and rosé wines (Table 3). However, the anthocyanin profiles of the spine wines consisted mainly of anthocyanidin diglucosides (Table 3). It should also be noted that malvidin-3,5-*O*-diglucoside appeared to be the major anthocyanin in the wines with the concentration of 189.06–1024.65 mg/L. It accounted for 58.29–78.10% of the total anthocyanin concentration in these wines.

**Table 2.** Retention time, maximum absorption wavelength (λ_max_), and mass spectrum of anthocyanins detected in wines.

Peak No.	Anthocyanin	Retention Time	λmax (nm)	Mass spectrum
1	Delphinidin-3,5-*O*-diglucoside	3.58	522	627, 465, 303
2	Cyanidin-3,5-*O*-diglucoside	4.10	-	611, 449, 287
3	Petunidin-3,5-*O*-diglucoside	4.67	523	641, 479, 317
4	Peonidin-3,5-*O*-diglucoside	6.17	525	625, 463, 301
5	Malvidin-3,5-*O*-diglucoside	7.95	524	655, 493, 331
6	Peonidin-3-*O*-(6-*O*-acetyl)-glucoside-5-*O*-glucoside	15.79	526	683, 521, 317
7	Malvidin −3-*O*-(6-*O*-acetyl)-glucoside-5-*O*-glucoside	18.18	526	697, 535, 493, 331
8	Delphinidin-3-*O*-(6-*O*-coumaroyl)-glucoside-5-*O*-glucoside	22.91	529	773, 611, 465, 303
9	Petunidin-3-*O*-(6-*O*-coumaroyl)-glucoside-5-*O*-glucoside	28.11	531	787, 625, 479, 317
10	Malvidin-3-*O*-(6-*O*-coumaroyl)-glucoside-5-*O*-glucoside	33.30	531	801, 639, 493, 331
11	Malvidin-3-*O*-(6-*O*-coumaroyl)-glucoside	42.79	532	639, 331

**Table 3.** Concentration of individual anthocyanins in wines.

Anthocyanin	W1	W2	W3	W4	W5	W6	W7	W8	W9	W10	W11
Delphinidin-3,5-*O*-diglucoside	5.79 ± 0.00	9.67 ± 0.03	34.58 ± 0.03	24.30 ± 0.01	2.85 ± 0.16	7.11 ± 0.01	6.63 ± 0.02	28.06 ± 0.02	4.76 ± 0.03	4.27 ± 0.04	5.81 ± 0.01
Cyanidin-3,5-*O*-diglucoside	0.22 ± 0.01	7.88 ± 0.04	11.14 ± 0.03	7.98 ± 0.00	1.85 ± 0.26	5.30 ± 0.05	-	-	3.76 ± 0.00	3.80 ± 0.03	4.02 ± 0.03
Petunidin-3,5-*O*-diglucoside	14.05 ± 0.21	16.84 ± 0.14	65.38 ± 0.07	47.60 ± 0.00	5.54 ± 1.59	15.46 ± 0.18	16.90 ± 0.01	58.90 ± 0.06	8.00 ± 0.02	9.58 ± 0.10	12.06 ± 0.04
Peonidin-3,5-*O*-diglucoside	-	9.81 ± 0.26	9.29 ± 0.06	7.53 ± 0.00	4.10 ± 2.23	8.62 ± 0.37	4.00 ± 0.04	1.47 ± 0.09	7.03 ± 0.04	-	-
Malvidin-3,5-*O*-diglucoside	189.06 ± 0.30	567.07 ± 1.41	1024.65 ± 0.20	739.43 ± 0.01	247.25 ± 2.32	518.93 ± 2.10	371.40 ± 0.05	754.77 ± 0.78	303.35 ± 0.20	395.45 ± 2.56	383.34 ± 0.59
Peonidin-3-*O*-(6-*O*-acetyl)-glucoside-5-*O*-glucoside	1.46 ± 0.04	2.76 ± 0.00	7.31 ± 0.01	5.90 ± 0.00	1.06 ± 0.00	4.45 ± 0.22	1.51 ± 0.15	5.21 ± 0.09	0.72 ± 0.01	1.30 ± 0.00	2.85 ± 1.61
Malvidin −3-*O*-(6-*O*-acetyl)-glucoside-5-*O*-glucoside	8.59 ± 0.36	26.45 ± 0.00	28.95 ± 0.10	14.04 ± 0.30	9.52 ± 0.08	11.91 ± 0.14	12.61 ± 0.01	30.09 ± 0.12	0.89 ± 0.01	10.77 ± 0.08	8.50 ± 0.04
Delphinidin-3-*O*-(6-*O*-coumaroyl)-glucoside-5-*O*-glucoside	4.29 ± 0.06	1.18 ± 0.01	28.27 ± 0.04	23.62 ± 0.23	-	5.68 ± 0.07	3.95 ± 0.22	25.98 ± 0.00	-	2.47 ± 0.09	4.29 ± 0.04
Petunidin-3-*O*-(6-*O*-coumaroyl)-glucoside-5-*O*-glucoside	6.46 ± 0.22	10.13 ± 0.06	40.19 ± 0.31	33.74 ± 1.17	3.68 ± 0.24	12.56 ± 0.42	9.67 ± 0.16	34.30 ± 1.04	1.95 ± 0.32	7.35 ± 0.06	8.96 ± 0.00
Malvidin-3-*O*-(6-*O*-coumaroyl)-glucoside-5-*O*-glucoside	64.04 ± 0.15	183.54 ± 0.12	294.63 ± 0.30	327.20 ± 2.66	94.36 ± 0.24	261.94 ± 0.21	132.07 ± 1.18	305.68 ± 2.93	52.71 ± 0.32	140.28 ± 0.53	148.14 ± 0.14
Malvidin-3-*O*-(6-*O*-coumaroyl)-glucoside	1.49 ± 0.01	3.15 ± 0.02	7.67 ± 0.01	7.44 ± 0.05	0.66 ± 0.02	5.48 ± 0.01	0.95 ± 0.02	7.13 ± 0.10	0.46 ± 0.02	1.21 ± 0.03	3.05 ± 0.00

Anthocyanin	W12	W13	W14	W17	W18
Delphinidin-3-*O*-diglucoside	0.22 ± 0.01	-	-	0.27 ± 0.01	0.79 ± 0.01
Cyanidin-3-*O*-diglucoside	-	-	-	0.30 ± 0.01	0.25 ± 0.01
Petunidin-3-*O*-diglucoside	0.29 ± 0.01	-	-	0.24 ± 0.01	1.01 ± 0.01
Peonidin-3-*O*-diglucoside	0.26 ± 0.01	-	0.26 ± 0.01	0.63 ± 0.14	0.46 ± 0.03
Malvidin-3-*O*-diglucoside	3.77 ± 0.02	0.77 ± 0.01	1.88 ± 0.01	0.78 ± 0.02	6.12 ± 0.01
Peonidin-3-*O*-(6-*O*-acetyl)-glucoside					0.84 ± 0.03
Malvidin-3-*O*-(6-*O*-acetyl)-glucoside	0.86 ± 0.02	-	-	0.25 ± 0.01	0.67 ± 0.06
Peonidin-3-*O*-(6-*O*-coumaroyl)-glucoside	0.25 ± 0.01	-	-	-	0.49 ± 0.01
Malvidin-3-*O*-(6-*O*-coumaroyl)-glucoside	0.63 ± 0.05	0.41 ± 0.01	0.28 ± 0.02	1.03 ± 0.01	0.64 ± 0.01

Data are mean ± standard deviation of duplicate tests. Quantitation was calculated using malvidin-3-*O*-glucoside; ‘-’ represents trace amount. White wines W15 and W16 were not studied.

Malvidin-3-*O*-(6-*O*-coumaryl)-glucoside-5-*O*-glucoside was observed to be the second highest level anthocyanin (64–327.20 mg/L) in these spine wines with the concentration representing 13.58–29.71% of the total anthocyanin concentration. In these spine wines, the wine sample W8 showed the highest level of this anthocyanin, whereas the lowest level was observed in the wine sample W9. It has been reported that malvidin-3-*O*-(6-*O*-acetyl)-glucoside represented the second highest level anthocyanin in red wines made of *V. vinifera* grape varieties [31]. However, wine sample W17 presented the highest concentration of malvidin-3-*O*-(6-*O*-coumaroyl)-glucoside, malvidin-3-*O*-diglucoside presented the second highest level anthocyanin, which was different with other *V. vinifera* grape wine. This may resulted from the different variety, vintage, and origin. Cyanidin-3,5-*O*-diglucoside, peonidin-3,5-*O*-diglucoside and delphinidin-3-*O*-(6-*O*-coumaroyl)-glucoside-5-*O*-glucoside were only detected in several spine wine samples and these wines showed the trace level of these anthocyanins.

It has been known that anthocyanins in wine mainly result from grapes since they are extracted from grape skin into wine during maceration and fermentation processes [13]. Spine grape have been reported to generally contain anthocyanidin diglucosides with 98.11–98.81% of the total anthocyanin content [23]. In our study, the concentration of the anthocyanidin diglucosides in these wines accounted for 96.64–98.65% of the total anthocyanin concentration, indicating that the anthocyanins in these spine wines were mainly derived from their spine grapes. It has been also reported that anthocyanidin-3,5-*O*-diglucosides was the predominant anthocyanin in wines produced by American non-*vinifera* grape cultivars and their hybrids [38]. Our result was also consistent with this report. Additionally, non-acylated anthocyanin derivatives have been reported to represent 59.64% of the total anthocyanin content in the skin of Xiangzhenzhu grape berries, whereas acylated anthocyanin derivatives only had 40.36% of the total anthocyanin content [23]. In this study, we found the similar observation that the total non-acylated anthocyanin derivative concentration in these wines was much higher than the concentration of the total acylated anthocyanin derivative level.

Anthocyanins display different structural forms with different color features in different pH conditions, including blue quinoidal base, red flavylium ion, colorless pseudo base/carbinol pseudo base, and pale yellow chalcone [39]. In wine, an equilibrium state exists in these different form anthocyanins. In the spine wines, anthocyanins tent to have a high percentage in quinoidal base form, resulting in the spine wines with a strong blue color. Besides, coumaroylated anthocyanidin diglucosides exhibited a higher level in these spine wines, which might further improve the blue hue of these wines, since coumaryol group has been suggested to favor the intramolecular copigmentation [40].

In order to investigate the effect of anthocyanins on color contribution of the spine wines, the PLSR was used (Supplementary Table 1). The partial regression coefficients indicated that all of the anthocyanins were negatively correlated to the L* value of these spine wines when the two factors were selected in this PLSR analysis. In the meantime, all the anthocyanins were suggested to show a negative correlation with b* value, but a positive correlation with a* and C values when the first factor was selected in the analysis. It should be noted that all the anthocyanins, except for cyaniding-3,5-*O*-diglucoside and peonidin-3,5-*O*-diglucoside, were negatively correlated to H value of these wine samples. The partial regression equations represented 89.1%, 78.7%, 78.5% and 78.7% of the total variance of the independent variable value of L*, a*, b*, and C, respectively. For the dependent variable value of L*, a*, b*, and C, it represented 86.1%, 52.9%, 33.7%, and 54.0% of the total variance.

When other variables are fixed, the partial regression coefficient represents an alteration degree of dependent variable along with a change of independent variable. Cyanidin-3,5-*O*-diglucoside, peonidin-3,5-*O*-diglucoside, peonidin-3-*O*-(6-*O*-acetyl)-glucoside-5-*O*-glucoside and malvidin-3-*O*-(6-*O*-coumaryl)-glucoside displayed higher partial regression coefficients (Supplementary Table 1). This indicated that these anthocyanins played more important roles in changing the color of the wine compared with other anthocyanins at the same concentration. It was observed that malvidin-3,5-*O*-diglucoside and malvidin-3-*O*-(6-*O*-coumaryl)-glucoside-5-*O*-glucoside had the lowest partial regression coefficients. However, these two anthocyanins contributed much greater to the wine color in the partial regression equations due to their level predominance in the wine samples. These results were consistent with the contribution of anthocyanidin monoglucosides to wine color [6,31].

### Total phenolic content and anthocyanins in spine wines

These spine wines had higher total phenolic content ranging from 951.84 to 3627.96 mg GAE/L compared with conventional wines (177.13–2007.92 mg GAE/L) (Table 4). The total phenolic content of white wine (W15, W16) and rosé wine (W12, W14) was not significant; however, the total phenolic content of spine rosé wine W1 was significantly higher. In red wines, W17 and W18 presented a similar total phenolic content compared with spine wines W7 and W10. The wine samples W3 and W4 contained the highest level of the total phenolic content. Among the spine wine samples, the wine samples W1, W5, and W9 displayed a similar level of phenolic content and they showed the lowest level. The total phenolic content in the other wine samples differed significantly. It has been reported that the total phenolic content of 24 wine samples made of *V. vinifera* grape varieties ranged from 1402 to 3130 mg GAE/L [15]. Another published study reported that *V. labrusca* grape wines produced in Brazil contained the total phenolic content of 1560–5015 mg GAE/L [41]. These indicated that spine wines might have the similar health benefits since its phenolic content was competitive with *V. vinifera* and *V. labrusca* grape wines.

**Table 4.** Total phenolic content and antioxidant activity of wines.

Wine samples	Total phenol content (mg GAE/L)	Total anthocyanin content (mg/L)	ABTS (µM)	DPPH (µM)	CUPRAC (µM)
W1	951.84 ± 24.08 g	299.17 ± 1.93	117.86 ± 4.55 g	75.83 ± 6.72 f	107.25 ± 6.89 hij
W2	2,092.58 ± 12.19 d	847.25 ± 6.39	370.86 ± 3.94c	203.92 ± 6.60 d	313.13 ± 4.60 d
W3	3,627.96 ± 24.50 a	1,570.11 ± 2.94	386.79 ± 1.01c	239.92 ± 1.18 c	474.88 ± 9.19 b
W4	3,477.77 ± 36.50 a	1,268.56 ± 9.55	458.00 ± 2.12 b	281.75 ± 10.14 a	493.75 ± 7.25b
W5	977.66 ± 36.45 g	375.43 ± 7.68	177.43 ± 3.94 f	108.08 ± 4.48 ef	145.50 ± 5.83 gh
W6	2,675.9 ± 60.77 c	881.59 ± 8.13	436.43 ± 5.56b	276.58 ± 3.77 ab	527.25 ± 12.55a b
W7	1,886.44 ± 12.07 e	577.27 ± 5.92	387.57 ± 3.94 c	220.42 ± 0.94cd	402.50 ± 5.48 c
W8	3,010.16 ± 48.34 b	1,274.00 ± 9.01	524.86 ± 3.13 a	276.42 ± 2.12ab	571.00 ± 9.02 a
W9	995.02 ± 12.38 g	388.44 ± 3.45	193.71 ± 1.11e f	88.00 ± 6.82f	138.5 ± 8.31g hi
W10	2,135.42 ± 72.65 d	584.33 ± 3.93	282.79 ± 11.11 d	132.92 ± 1.18e	237.13 ± 12.02 e
W11	1,440.95 ± 60.83 f	585.85 ± 0.95	223.36 ± 2.42 e	125.33 ± 4.12 e	171.88 ± 0.35f g
W12	299.86 ± 2.01i	6.27 ± 0.10	31.35 ± 0.01 h	25.74 ± 0.11 g	80.13 ± 2.52 ijkl
W13	745.24 ± 76.19 h	1.17 ± 0.01	31.21 ± 0.02 h	27.43 ± 0.29g	103.02 ± 0.65h ijk
W14	177.13 ± 13.07 i	2.29 ± 0.14	27.83 ± 0.26 h	7.13 ± 0.26 g	39.99 ± 0.01 k l
W15	262.32 ± 8.64 i	-	31.25 ± 1.14 h	17.15 ± 0.01 g	56.26 ± 1.25j kl
W16	204.57 ± 7.94 i	-	26.87 ± 33.10 h	12.16 ± 0.04 g	38.49 ± 0.48 l
W17	2007.92 ± 107.80 de	3.11 ± 0.11	448.17 ± 24.88b	241.97 ± 15.19 c	227.25 ± 41.00 ef
W18	1961.67 ± 52.11 de	11.25 ± 0.07	475.43 ± 17.14 b	247.86 ± 15.86 bc	188.96 ± 14.63 efg

Different letters in each column indicate significant differences at *p* ≤ 0.05; DPPH, ABTS, and CUPRAC activity are expressed as µM trolox equivalent; the volume of wine samples in antioxidant activity assays is 0.1 mL after 30-time dilution using 15% (v/v) ethanol. **‘-’**: Total anthocyanin content was not detected in white wines W15 and W16.

The spine wine samples also showed the high level of the total anthocyanin content (Table 4). A published study reported that the total anthocyanin content in spine wine made of spine grape Junzi ^#^1, Junzi ^#^2, Longfeng, and Yishan ranged at 90–260 mg/L and the differences were explained by the differences on grape strain, growth origin and winemaking technology [12,14,28,37,42]. Spine grape strain Xiangzhenzhu has also been reported to possess the high total anthocyanin content (16 mg/g dry skin weight) [23]. In our study, the spine wines (W3) produced from the spine grape strain of Xiangzhenzhu1^#^ exhibited higher level of the total anthocyanin content. No significant differences on the total anthocyanin content were observed between the wine sample W4 and W8. The similar levels of the total anthocyanins were also observed in the wine samples W7, W10, and W11. The *V. vinifera* grape wines in this study presented the lowest level of total anthocyanin content, which resulted from their older vintage.

### Antioxidant activity of spine wines

In the ATBS assay, the white (W15 and W16) and rosé wines (W12, W13 and W14) made of *V. vinifera* grape presented the lowest TEAC value; however, the rosé wine (W1) made of spine grape significantly presented higher TEAC value. In red wines, the highest TEAC value was observed in the sample W8. The spine wines W2, W3, W4, W6, and W7 and W17, W18 exhibited higher TEAC value. These indicated that the antioxidants in these wines worked mainly as hydrogen donors to scavenge the oxidations. The other wine samples showed the relatively low TEAC value probably due to their low level of the antioxidants. In the DPPH analysis, the wine samples W4, W6, and W8 showed the highest TEAC value, followed by the sample W3, W7 and the sample W17, W18. The white (W15 and W16) and rosé wines (W12, W13, and W14) made of *V. vinifera* grape also presented the lowest TEAC value. The wine sample W1 and W9 displayed the higher TEAC value than white (W15 and W16) and rosé wine (W12, W13 and W14).

The TEAC value profile in the CUPRAC measurement was similar to that in ABTS and DPPH analysis.

The wine sample W6, W8 exhibited the highest TEAC value in the CUPRAC measurement, followed by the wine sample W4 and W3. The lowest CUPRAC TEAC value was observed in samples W12–W16. These spine wines were fermented from different strains of spine grapes and their winemaking process might have the difference. Therefore, their antioxidant activity showed the variations [15,20,21,42]. It has been speculated that phenolic compounds are the major antioxidants in wine. It should also be noted that other compounds in red wines could also exert as antioxidants to provide wine with antioxidant properties, such as peptides, polysaccharides, tartaric esters, and minerals [43].

### Correlation between antioxidant activity and phenolic compounds in spine wines

In order to investigate whether or not the phenolic compounds in the spine wine contributed to the antioxidant properties of spine wine, correlation analyses were conducted (Table 5). The results showed that a significantly positive correlation existed between the antioxidant activity of the spine wines and the total phenol or the total anthocyanins, indicating the antioxidants of the spine wines resulted mainly from these phenolic compounds, including anthocyanins. These results were consistent with previously published reports [44,45]. Additionally, the coefficients in correlation between the antioxidant activity of the spine wines and the total phenol was higher than that between the antioxidant activities and the total anthocyanins, indicating that non-anthocyanin phenolic compounds in these spine wines also played significant roles in contribution of the wine antioxidant activity. On the other hand, malvidin-3-*O*-(6-*O*-coumaroyl)-glucoside-5-*O*-glucoside had a higher coefficient with these antioxidant measurements compared to malvidin-3,5-*O*-diglucoside. This suggested that malvidin-3-*O*-(6-*O*-coumaroyl)-glucoside-5-*O*-glucoside might have a higher antioxidant capacity than malvidin-3,5-*O*-diglucoside. It has been suggested that antioxidant capacity of antioxidants essentially relies on their chemical structure and concentration [46]. Meanwhile, anthocyanin acylation has been reported to improve the antioxidant feature of anthocyanins [42,46]. In the present study, malvidin-3-*O*-(6-*O*-coumaroyl)-glucoside-5-*O*-glucoside, compared to malvidin-3,5-*O*-diglucosides, had one more phenolic hydroxyl group, which could enhance the antioxidant function.

**Table 5.** Correlations of antioxidant activity of spine wines with phenolic compounds in spine wines.

Correlation	ABTS	DPPH	CUPRAC	Total phenol	Total anthocyanin	Malvidin-3,5-*O*-diglucosides	Malvidin-3-O-(6-O-coumaroyl)-glucoside-5-glucoside
ABTS	1						
DPPH	0.972**	1					
CUPRAC	0.970**	0.984**	1				
Total phenol	0.868**	0.891**	0.899**	1			
Total anthocyanin	0.822**	0.841**	0.861**	0.957**	1		
Malvidin-3,5-*O*-diglucosides	0.778**	0.790**	0.814**	0.934**	0.992**	1	
Malvidin-3-O-(6-O-coumaroyl)-glucoside-5-glucoside	0.899**	0.930**	0.932**	0.962**	0.949**	0.906**	1

**Represents significant difference at 0.01 level.

## Conclusions

In conclusion, the young spine wines had the purple-red color with the strong blue tone. These wines were rich in phenolic compounds, including anthocyanidin diglucosides. Malvidin-3,5-*O*-diglucoside and malvidin-3-*O*-(6-*O*-coumaroyl)-glucoside-5-*O*-glucoside were the predominant anthocyanins in these wines. Spine wines W3, W4, W6, and W8 exhibited high level of antioxidant activity estimated by ABTS, DPPH, and CUPRAC analyses. The high antioxidant capacity of the spine wine mainly resulted from phenolic compounds, including anthocyanins. Malvidin-3-*O*-(6-*O*-coumaroyl)-glucoside-5-*O*-glucoside showed higher antioxidant property that malvidin-3,5-*O*-diglucosides.

## Supplementary Material

Supplementary_table.doc
