# Supplementary material for: Color, anthocyanin, and antioxidant characteristics of young wines produced from spine grapes (Vitis davidii Foex) in China
Source: Food Nutr Res. 2017 Jul 14;61(1):1339552. doi: 10.1080/16546628.2017.1339552 (PMC5533146; doi:10.1080/16546628.2017.1339552)
Supplement: Supplementary_table.doc [file zfnr_a_1339552_sm5002.doc]

**Supplementary Table 1**. Partial regression coefficients between anthocyanins and spine wine color

| Anthocyanin | L*^1^ | a* | b* | C | H |
| --- | --- | --- | --- | --- | --- |
| Constant | 82.622 | 37.882 | -4.027 | 38.485 | -6.626 |
| Delphinidin-3,5-*O*-diglucoside | -0.039 | 0.085 | -0.026 | 0.087 | -0.018 |
| Cyanidin-3,5-*O*-diglucoside | -0.528 | 0.239 | -0.043 | 0.242 | 0.032 |
| Petunidin-3,5-*O*-diglucoside | -0.017 | 0.043 | -0.014 | 0.044 | -0.012 |
| Peonidin-3,5-*O*-diglucoside | -0.745 | 0.198 | -0.002 | 0.198 | 0.118 |
| Malvidin-3,5-*O*-diglucoside | -0.01 | 0.005 | -0.001 | 0.005 | 0.001 |
| Peonidin-3-*O*-(6-*O*-acetyl)-glucoside-5-*O*-glucoside | -0.575 | 0.5 | -0.201 | 0.518 | -0.19 |
| Malvidin -3-*O*-(6-*O*-acetyl)-glucoside-5-*O*-glucoside | -0.304 | 0.109 | -0.023 | 0.109 | -0.012 |
| Delphinidin-3-*O*-(6-*O*-coumaryl)-glucoside-5-*O*-glucoside | -0.007 | 0.083 | -0.034 | 0.085 | -0.034 |
| Petunidin-3-*O*-(6-*O*-coumaryl)-glucoside-5-*O*-glucoside | -0.076 | 0.077 | -0.027 | 0.079 | -0.024 |
| Malvidin-3-*O*-(6-*O*-coumaryl)-glucoside-5-*O*-glucoside | -0.038 | 0.013 | -0.005 | 0.013 | -0.003 |
| Malvidin-3-*O*-(6-*O*-coumaryl)-glucoside | -0.637 | 0.391 | -0.166 | 0.406 | -0.166 |
| R^2^ | 0.861 | 0.529 | 0.337 | 0.54 | 0.132 |
| Total variance of independent variables (%) | 89.1 | 78.7 | 78.5 | 78.7 | 71.3 |
| Total variance of dependent variables (%) | 86.1 | 52.9 | 33.7 | 54 | 13.2 |

^1^ two factors in PLSR analysis was selected since all the anthocyanins were negatively correlated to L* value.
